# Numerical Analysis of the Influence of Air Flow Rate on the Development of the Porous Structure of Activated Carbons Prepared from Macadamia Nut Shells

**DOI:** 10.3390/ma17246264

**Published:** 2024-12-21

**Authors:** Mirosław Kwiatkowski, Guojie Zhang

**Affiliations:** 1 Faculty of Energy and Fuels, AGH University of Krakow, al. Adama Mickiewicza 30, 30-059 Krakow, Poland; 2 State Key Laboratory of Clean and Efficient Coal Utilization, College of Chemical Engineering and Technology, Taiyuan University of Technology, Taiyuan 030024, China; zhangguojie@tyut.edu.cn; 3 Key Laboratory of Coal Science and Technology, Ministry of Education, Taiyuan University of Technology, Taiyuan 030024, China

**Keywords:** activated carbons, nitrogen, carbon dioxide, porous structure, air flow rate

## Abstract

This paper presents the numerical analysis of the influence of air flow rate on the porous structure development of activated carbons prepared from macadamia nut shells. The analyses based on nitrogen and carbon dioxide isotherms were carried out by the new numerical clustering-based adsorption analysis method. Therefore, it was possible to evaluate the porous structure with high precision and reliability. In particular, the results obtained showed that activated carbon prepared at an air flow rate of 700 cm^3^/min has the highest adsorption capacity with respect to this adsorbate, but with surface heterogeneity. On the other hand, numerical analysis based on carbon dioxide adsorption isotherms showed that the activated carbon with the highest adsorption capacity towards carbon dioxide is the sample obtained at an air flow rate of 500 cm^3^/min. The analyses conducted have shown that too high an air flow rate causes a violent oxidation reaction, leading to uncontrolled burning of the carbonaceous substance and destruction of the structure of the smallest micropores.

## 1. Introduction

One of the largest problems of the contemporary world, which has a significant impact on global climate change, is the observed gradual increase in the average temperature of the Earth’s atmosphere. This undesirable effect is caused by excessive emissions of greenhouse gases, particularly carbon dioxide (CO_2_), and the accumulation of these gases in the upper atmosphere [1]. The widespread burning of fossil fuels is largely responsible for anthropogenic CO_2_ emissions [2,3]. Consequently, numerous initiatives and measures are being taken to reduce CO_2_ emissions, of which the capture and use of this gas is widely recognised today as one of the most effective solutions for reducing its emissions into the atmosphere [4]. 

Among the many methods for capturing CO_2_, the most well-known are absorption [5,6] and adsorption techniques [7]. However, the method of adsorption on the surface of solids is very promising due to its high efficiency, widespread availability, and ease of regeneration of adsorbents, as well as the relatively low energy consumption needed for the process [7]. In contrast, of the many adsorbents used in CO_2_ capture processes, such as organometallic compounds [8], carbon foams [9], carbon aerogel fibres [10], and activated carbons (AC) with a highly developed specific surface area and microporous structure, are the most optimal under industrial conditions [11,12,13,14].

Activated carbons are most often produced by physical or chemical activation in which a microporous structure is developed [15,16]. Physical activation is mostly carried out as a two-step process that involves the carbonisation of an organic material with a high carbon content, followed by the activation of the resulting char at a high temperature, i.e., 700–1000 °C, in the presence of suitable oxidising gases such as carbon dioxide, oxygen, steam, or mixtures thereof [17]. The physical activation is a most popular method for obtaining activated carbons on an industrial scale due to the uncomplicated nature of the process and the simple design of the equipment used, the rapid process time, and the common availability of raw materials, despite the average development of the porous structure of the carbonaceous materials obtained [18]. It should be noted, however, that the physical activation process, because it is carried out at high temperatures, involves high energy costs [19].

The chemical activation process instead involves the direct treatment of an organic material or charcoal by impregnation with a chemical activating agent, such as potassium hydroxide, potassium carbonate, sodium carbonate, magnesium chloride, phosphoric acid, sulphuric acid, aluminium chloride, sodium chloride, or zinc chloride, followed by heating without air at temperatures of approximately 400–800 °C [20]. In the chemical activation, the process is conducted at lower temperatures compared to the physical activation process. Because activating agents act as dehydrating agents that inhibit the formation of tar and volatiles during the process, the yield of porous activated carbon can be increased, and the activation temperature and time can be reduced, likened to the physical activation [21,22].

As an alternative to the one-step chemical activation process, a two-step process can be used, whereby the raw material is first carbonised to produce charcoal, then impregnated with a chemical activating agent, and finally activation of the impregnated char at high temperature is carried out to produce activated carbon. The two-stage process has the advantage of providing an increased elemental carbon content in the final product, with the first carbonisation stage producing an initial porosity that is further developed by chemical activation [23].

The quantity of activator, the mass ratio of activator to raw material, and the reaction temperature are among the most significant parameters affecting the formation of the porous structure in the production of activated carbons by chemical activation [24]. It should also be noted that, in contrast to physical activation, chemical activation, which is carried out at much lower temperatures, can provide products with greater porous structure development and higher yields [25]. The properties of activated carbons are also highly dependent on the raw material. Most commercially available activated carbons are made from fossil fuel-based precursors, but there is an increasing emphasis on obtaining activated carbons from biomass waste materials, which are cheaper, readily available, renewable, structurally porous, and environmentally friendly [26,27,28,29,30].

As a result of the ever-increasing demands placed on the adsorption technique used in carbon dioxide capture processes, new methods of producing activated carbons and primary raw materials for their manufacture, as well as methods of modifying their adsorption properties, are being sought. Some of the most promising methods for producing activated carbons involve the use of molten inert salts [31].

## 2. Materials and Methods

The paper [32] proposes an original method to produce activated carbons (AC) from macadamia nut shells in a one-step process by pyrolysis in an air atmosphere using molten potassium chloride (KCl). In the aforementioned study, a group of activated carbons was obtained by mixing macadamia nut shells with KCl and melamine in deionised water, conducting the mixing process for 5 h. KCl, and melamine were purchased from Tianjin Kermel Chemical Reagent Company Limited.

The resulting mixture was then dried in an oven at 383 K, and the dried material was pyrolyzed at 973 K in an air stream at different air flow rates, i.e., 200, 500, and 700 cm^3^/min for 2 h. Obtained samples of activated carbons were labelled as uAC, where u denoted the air flow rate (cm^3^/min). Nitrogen adsorption isotherms were measured with Beishide PS2000 for the obtained activated carbon samples, from which, among other things, the specific surface area of *S*_BET_ was calculated via the BET method [33], the total pores volume *V*_T_ at *P*/*P*_0_ = 0.99, and the micropores volume *V_o_* via the t-plot method [34]. 

The methods used in the mentioned research are, however, criticised for excessive simplifying assumptions that do not correlate with reality, including failure to take into account surface heterogeneity, as well as underestimation of pores size [35]. As a result of the growing demands placed on adsorbents, as already mentioned, techniques for their manufacture are being upgraded, but this requires a detailed valuation of the porous structure with particular regard to surface heterogeneity, which is not provided by the methods used in this study. Therefore, the concept of carrying out new reliable analyses of the determined adsorption isotherms using an advanced numerical method of clustering-based adsorption process analysis on heterogeneous surfaces (LBET) was developed [36,37,38,39,40].

The LBET method, which has been designated in detail in previous work [36,37,38,39,40], is based on a group of unique mathematical models of the adsorption process on the heterogeneous surfaces of carbonaceous adsorbents. The models mentioned have five parameters, i.e., the volume of the first adsorbed layer *V_hA_*, the dimensionless energy parameter for the first adsorbed layer *Q_A_*/*RT*, the energy parameter for the higher adsorbed layers *B_C_*, the geometrical parameter determining the height of the adsorbate molecule clusters *α*, and the geometrical parameter of the porous structure determining the width of the adsorbate molecule clusters *β*, obtained by fitting LBET models to an empirical adsorption isotherm [36,37,38,39,40]. A fast numerical procedure for multivariate fitting was implemented in the LBET method, allowing the determination of the value of the surface heterogeneity parameter *h* and the shape of the adsorption energy distribution on the adsorbent surface.

## 3. Results

**Figure 1:**
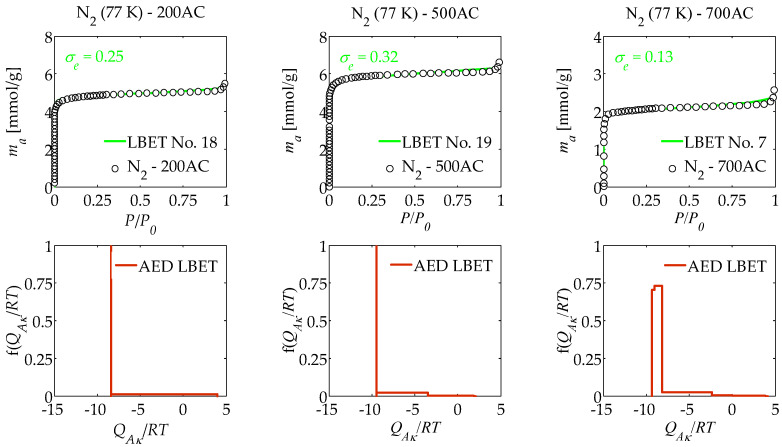
The results of the analysis of a porous structure of activated carbons via the LBET method, based on nitrogen adsorption isotherms determined at 77 K, where AED LBET is the adsorption energy distribution.

**Figure 2:**
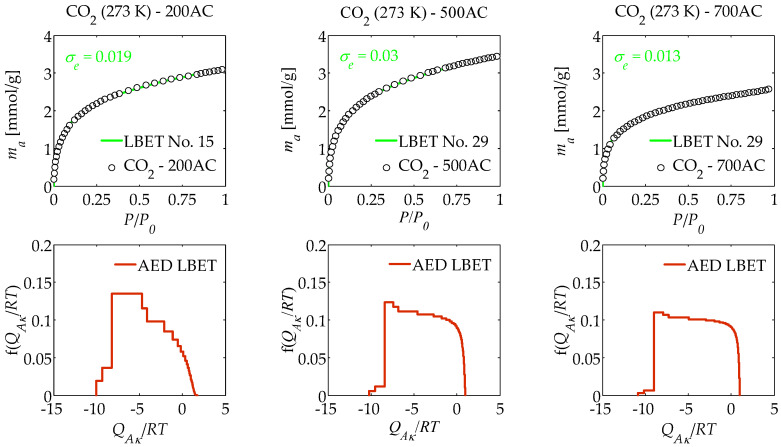
Same as Figure 1, but presenting the results of the analysis of a porous structure of activated carbons, based on carbon dioxide adsorption isotherms determined at 273 K.

**Figure 3:**
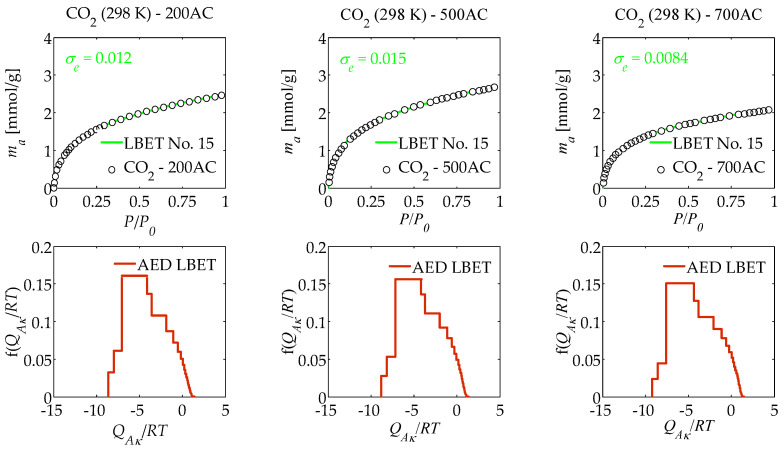
Same as Figure 1, but presenting the results of the analysis of a porous structure of activated carbons, based on carbon dioxide adsorption isotherms determined at 298 K.

The results of the numerical analysis of the influence of air flow rate on the porous structure development of activated carbons prepared from macadamia nut shells in a one-step process by pyrolysis in an air atmosphere using molten potassium chloride (KCl) at different air flow rates, i.e., 200, 500, and 700 cm^3^/min based on nitrogen (N_2_) adsorption isotherms determined at 77 K, and carbon dioxide (CO_2_) isotherms determined at 273 K and 298 K using the LBET method, are summarised in Table 1, Table 2 and Table 3 and shown in Figure 1, Figure 2 and Figure 3.

The results of the analyses carried out on the basis of nitrogen adsorption isotherms via the LBET method, summarised in Table 1 and Figure 1 for the 200AC sample, i.e., obtained at an air flow rate of 200 cm^3^/min, showed that this material is characterised by nitrogen cluster growth limitations related to geometrical pore limitations, as indicated by the number of the best-fitted LBET model. The value of the *V_hA_* parameter determined for the 200AC activated carbon sample indicates a very small volume of the first adsorbed layer (*V_hA_* = 0.161 cm^3^/g), and the values of the geometrical parameters *α* and *β* indicate, respectively, that very low and significantly branching clusters of nitrogen molecules are formed in the pores of the analysed material. On the basis of the calculated values of the energy parameters *Q_A_*/*RT* and *B_C_*, it can be concluded that on the surface of the 200AC sample there are favourable conditions for the multilayer adsorption process (*Q_A_*/*RT* = −8.42, *B_C_* = 3.94). The value of the parameter *h* indicates that the surface of the analysed material is characterised by a low degree of heterogeneity (*h* = 2).

The shape of the adsorption energy distribution (AED LBET) determined for 200AC activated carbon (see Figure 1) shows that there is a predominant proportion of primary adsorption sites on the surface of this material with nearly equal adsorption energy and a small number of primary adsorption sites with a wider adsorption energy distribution.

The results of analyses via the LBET method obtained for activated carbon 500AC, i.e., prepared in air flow rate equal to 500 cm^3^/min, showed that this material is characterised by limitations in the growth of clusters of nitrogen molecules related to geometric limitations of pores, as indicated by the number of the best-fitted LBET model, i.e., 19. The value of the parameter *V_hA_* (*V_hA_* = 0.179 cm^3^/g) indicates a small volume of the first adsorbed layer, and the values of the geometric parameters *α* and *β* indicate, respectively, that in the pores of the analysed sample of activated carbon, low, slightly branching clusters of adsorbate molecules are formed (*α* = 0.14 and *β* = 1.26). Based on the values of energy parameters *Q_A_*/*RT* and *B_C_*, i.e., for the first adsorbed layer and the subsequent layers, it can be concluded that the energy conditions for only a single-layer adsorption process occur on the surface of the 500AC activated carbon (*Q_A_*/*RT* = −1.35 and *B_C_* = 1.00).

The value of the parameter *h* shows that the surface of the analysed 500AC activated carbon is heterogeneous (*h* = 3), and the shape of the adsorption energy distribution on the surface of the 500AC activated carbon shows, as in the case of the 200AC sample, a dominant share of sites with equal adsorption energy. However, the 500AC sample returns a higher proportion of adsorption sites with a wider adsorption energy distribution.

The last sample analysed was activated carbon obtained at an air flow rate equal to 700 cm^3^/min, designated as 700AC. This material was characterised by limits on the growth of nitrogen molecule clusters related to the competitive expansion of neighbouring clusters. The very high value of the *V_hA_* parameter obtained for this activated carbon, i.e., *V_hA_* = 0.642 cm^3^/g, is noteworthy, being considerably higher than the corresponding parameters determined for samples obtained at lower air flow rates, i.e., 200 cm^3^/min and 500 cm^3^/min, in turn. Low and significantly branching clusters of nitrogen molecules form in the pores of the 700AC activated carbon, as indicated by the values of the geometrical parameters (*α* = 0.18 and *β* = 3.33). The values of the adsorption energy parameters (*Q_A_/RT* = −9.33 and *B_C_* = 7.73) showed preferential conditions for multilayer adsorption to occur on the surface of this sample. Note the significant value of the surface heterogeneity parameter (*h* = 5), which indicates that the surface of the analysed material is highly heterogeneous. On the other hand, the adsorption energy distribution (AED LBET) on the surface of the analysed 700AC activated carbon, determined by the LBET method and shown in Figure 1, indicates a predominant share of sites with a narrow range of adsorption energy and a much smaller share of sites with a wider range of adsorption energy.

In the case of the sample obtained at an air flow rate equal to 700 cm^3^/min, a significantly larger volume of the first adsorbed layer was observed compared to samples obtained at lower temperatures, but with an increase in the degree of surface heterogeneity. This is due to the fact that the high air flow rate favours the formation of micropores, thanks to the rapid removal of the carbon decomposition products from the surface. Note the high reliability of the results obtained, as indicated by the values of the parameters *σ_e_* and *w_id_*, i.e., the dispersion value of the fitting error and the adsorption system identifiability index, respectively.

Analysis of the porous structure and adsorption properties of the tested activated carbons based on the carbon dioxide adsorption isotherms determined at 273 K via the LBET method (see Table 2) showed that in the case of the 200AC sample, the limitations on clusters of CO_2_ molecules expansion are due to the competitive expansion of neighbouring clusters, while in the case of the 500AC and 700AC activated carbons, the limitations on clusters of CO_2_ molecules expansion are due to geometrical pore limitations. The *V_hA_* parameter values determined for the samples analysed indicate a small volume of the first adsorbed layer, with the highest *V_hA_* parameter value obtained for the 500AC activated carbon and the lowest for 700AC. The determined values of the geometrical parameters *α* and *β* showed that very high and significantly branching clusters of carbon dioxide molecules form in the pores of the analysed samples.

The parameters *Q_A_*/*RT* and *B_C_* obtained for the analysed samples on the basis of adsorption isotherms indicate strongly preferential conditions for the monolayer adsorption process to occur. In turn, it can be concluded from the values of the *h* parameters that the surface of the analysed samples is strongly heterogeneous with respect to carbon dioxide.

From the shapes of the adsorption energy distribution (AED LBET) on the surface of the analysed activated carbons, it can be concluded that there is a wide range of adsorption energy of primary adsorption sites on the surface. However, in the case of the activated carbon sample obtained at an air flow rate equal to 200 cm^3^/min, a predominant proportion of high-energy adsorption sites can be observed compared to the other two activated carbons analysed (Figure 2).

As part of this study, an analysis was also carried out on the basis of carbon dioxide adsorption isotherms determined at temperature 298 K. The results of the calculations are summarised in Table 3, and the adsorption isotherms and best-fitted LBET class models, as well as the adsorption energy distribution (AED LBET) on the surface of the analysed activated carbons, are shown in Figure 3. 

In the case of an analysis of the results obtained for adsorption isotherms determined at 298 K, it should be borne in mind that the results obtained provide information on the adsorption process under these conditions and are not a sound basis for drawing reliable conclusions, particularly about the microporous structure of the materials on which this process occurs.

As can be seen from the results summarised in Table 3 for the analysis of carbon dioxide isotherms determined at 298 K, for all the activated carbons analysed, the limitations on the growth of clusters of carbon dioxide molecules are due to the competitive expansion of neighbouring clusters. In turn, the values of the *V_hA_* parameters indicate that the analysed activated carbons are characterised by a small volume of the first adsorbed layer, with the largest volume of the first adsorbed layer obtained for 500AC activated carbon and the smallest for 700AC activated carbon.

The values of geometrical parameters *α* and *β* determined for the analysed activated carbons suggest that high and significantly branching clusters of carbon dioxide molecules form in their pores, and the values of the energy parameters *Q_A_*/*RT* and *B_C_* indicate that energy conditions favouring only monolayer adsorption occur on the surface of these materials. Note that the surface of all analysed samples is strongly heterogeneous, as indicated by the values of the *h* parameter (*h* = 9). 

The shape of the AED LBET, i.e., adsorption energy distribution determined from carbon dioxide adsorption isotherms obtained at temperature 293 K via the LBET method, shows a wide range of adsorption energies on the surface of the analysed samples with a significant proportion of high-energy adsorption sites.

## 4. Conclusions

This paper presents the original results of a numerical analysis of the effect of air flow rate on the formation of the porous structure of activated carbons prepared from macadamia nut shells in a one-step process by pyrolysis in an air atmosphere using molten potassium chloride at different air flow rates. The analyses mentioned were based on nitrogen adsorption isotherms determined at 77 K, and carbon dioxide isotherms determined at 273 K and 298 K using the new numerical clustering-based adsorption analysis method.

Analyses based on nitrogen adsorption isotherms showed that activated carbon obtained at an air flow rate of 700 cm^3^/min has the highest adsorption capacity with respect to this adsorbate, but with significant surface heterogeneity. On the other hand, analysis based on carbon dioxide adsorption isotherms determined at 273 K showed that the activated carbon with the highest adsorption capacity is the sample obtained at an air flow rate of 500 cm^3^/min, noting, however, the very high surface heterogeneity for carbon dioxide adsorption, which may be undesirable in many industrial adsorption processes. The results of an analysis of the adsorption process based on adsorption isotherms determined at 298 K are also presented, which indicated that the temperature of the adsorption process has a significant effect on its course.

As demonstrated in the conducted studies, the air flow supports the oxidation of the reactive carbonaceous substance, thereby developing microporosity. However, excessively high air flow velocities can exacerbate the uncontrolled oxidation reaction, leading to the uncontrolled burning of the carbon material and the destruction of the smallest micropore structures. This effect was observed in the case of the 700AC activated carbons, as evidenced by the analysis of carbon dioxide adsorption isotherms determined for the tested activated carbons.

## Figures and Tables

**Table 1:** The results of the analysis of a porous structure of activated carbons via the LBET method, based on nitrogen adsorption isotherms determined at 77 K.

AC	Model No.	*V_hA_* [cm^3^/g]	*α*	*β*	*Q_A_*/*RT*	*B_C_*	*h*	*σ* *_e_*	*w_id_*
200AC	18	0.161	0.14	2.37	−8.42	3.94	2	0.25	0.81
500AC	19	0.179	0.14	1.26	−9.48	1.00	3	0.32	0.47
700AC	7	0.642	0.18	3.33	−9.33	7.73	5	0.13	0.52

Where AC is the analysed activated carbon; Model No. is the number of the best-fitted model of the LBET class; *V_hA_* is the volume of the first adsorbed layer; *α* is the geometrical parameter of the porous structure determining the height of the adsorbate molecule clusters; *β* is the geometrical parameter of the porous structure determining the width of the adsorbate molecule clusters; *Q_A_*/*RT* is the dimensionless energy parameter for the first adsorbed layer; *B_C_* is the dimensionless adsorption energy parameter for the higher adsorbed layers; *h* is the surface heterogeneity parameter; *σ_e_* is the dispersion value of the fit error; and *w_id_* is the adsorption system identifiability index.

**Table 2:** The results of the analysis of a porous structure of activated carbons via the LBET method, based on carbon dioxide adsorption isotherms determined at 273 K.

AC	Model No.	*V_hA_* [cm^3^/g]	*α*	*β*	*Q_A_*/*RT*	*B_C_*	*h*	*σ* *_e_*	*w_id_*
200AC	15	0.211	0.90	5.11	−10.03	2.07	9	0.019	0.43
500AC	29	0.246	0.98	2.65	−10.22	1.00	9	0.03	0.19
700AC	29	0.185	0.99	2.22	−10.89	1.00	9	0.013	0.21

**Table 3:** The results of the analysis of a porous structure of activated carbons via the LBET method, based on carbon dioxide adsorption isotherms determined at 298 K.

AC	Model No.	*V_hA_* [cm^3^/g]	*α*	*β*	*Q_A_*/*RT*	*B_C_*	*h*	*σ* *_e_*	*w_id_*
200AC	15	0.170	0.85	4.89	−8.66	1.51	9	0.012	0.81
500AC	15	0.184	0.87	5.20	−8.80	1.41	9	0.015	0.73
700AC	15	0.143	0.89	4.74	−9.25	1.63	9	0.0084	0.65

## Data Availability

The original contributions presented in the study are included in the article, further inquiries can be directed to the corresponding author.
